# Zika RNA and Flavivirus-Like Antigens in the Sperm Cells of Symptomatic and Asymptomatic Subjects

**DOI:** 10.3390/v13020152

**Published:** 2021-01-21

**Authors:** Hernan Vanegas, Fredman González, Yaoska Reyes, Edwing Centeno, Jayrintzina Palacios, Omar Zepeda, Marie Hagbom, Matthew H. Collins, R. Matthew Coward, Sylvia Becker-Dreps, Natalie Bowman, Filemón Bucardo

**Affiliations:** 1 Department of Microbiology, Faculty of Medical Science, National Autonomous University of Nicaragua, León 21000, Nicaragua; luis_12vanegas@yahoo.es (H.V.); fredman.gonzalez@cm.unanleon.edu.ni (F.G.); yaoska.reyes@cm.unanleon.edu.ni (Y.R.); eduing42@hotmail.com (E.C.); jayrintzina.palacios@cm.unanleon.edu.ni (J.P.); zepeomi@gmail.com (O.Z.); 2 Division of Molecular Virology, Department of Clinical and Experimental Medicine, Linköping University, SE-581 83 Linköping, Sweden; marie.hagbom@liu.se; 3 Hope Clinic of the Emory Vaccine Center, Division of Infectious Diseases, Department of Medicine, School of Medicine, Emory University, Decatur, GA 30030, USA; matthew.collins@emory.edu; 4 Department of Urology, University of North Carolina at Chapel Hill, Chapel Hill, NC 27599, USA; mcoward@med.unc.edu; 5 Departments of Family Medicine and Epidemiology, University of North Carolina at Chapel Hill, Chapel Hill, NC 27599, USA; sbd@email.unc.edu; 6 Division of Infectious Diseases, University of North Carolina at Chapel Hill, Chapel Hill, NC 27599, USA; natalie_bowman@med.unc.edu

**Keywords:** Zika antigens, asymptomatic, sperm cells, semen, immunofluorescence

## Abstract

Zika virus (ZIKV) RNA has been found to remain in human semen for up to one year after infection, but the presence of Flavivirus antigens in the different compartments of semen has been largely unexplored. Following the introduction of ZIKV in Nicaragua (2016), a prospective study of patients with clinical symptoms consistent with ZIKV was conducted in León to investigate virus shedding in different fluids. ZIKV infection was confirmed in 16 male subjects (≥18 years of age) by RT-qPCR in either blood, saliva or urine. Of these, three provided semen samples at 7, 14, 21, 28, 60 and 180 days postsymptom onset (DPSO) for Flavivirus antigens and RNA studies. These cases were compared with 19 asymptomatic controls. Flavivirus antigens were examined by immunofluorescence (IF) using the 4G2 Mabs, and confocal microscopy was used to explore fluorescence patterns. The three (100%) symptomatic subjects and 3 (16%) of the 19 asymptomatic subjects had Flavivirus antigens and viral RNA in the spermatozoa fraction. The percentage of IF Flavivirus-positive spermatozoa cells ranged from 1.9% to 25% in specimens from symptomatic subjects, as compared with 0.8% to 3.8% in specimens from asymptomatic controls. A marked IF-pattern in the cytoplasmic droplets and tail of the spermatozoa was observed. The sperm concentrations (45 × 10^6^/mL vs. 63.5 × 10^6^/mL, *p* = 0.041) and the total motility percentage (54% vs. 75%, *p* = 0.009) were significantly lower in specimens from ZIKV-positive than in those of ZIKV-negative. In conclusion, this study demonstrated the presence of Flavivirus antigens and RNA within a time frame of 28 DPSO in sperm cells of symptomatic and asymptomatic subjects during the ZIKV epidemic. These findings have implications for public health, in terms of nonarthropod-born, silent transmission facilitated by sperm cells and potential transmission from asymptomatic males to pregnant women, with consequences to the fetus.

## 1. Introduction

Zika virus (ZIKV) is a mosquito-borne flavivirus that typically causes no symptoms or a mild disease characterized by fever, rash, conjunctivitis and arthralgia, lasting for 2–7 days [1]. Zika has caused significant concern in recent years with the discovery that it targets the neural progenitor cells in developing fetuses, disrupting neural development and resulting in congenital malformations and lifelong disabilities [2]. Moreover, studies in animal models have implicated the proximal male reproductive tract (i.e., testis and epididymis) as a target of ZIKV infection [3,4], and human ex-vivo tissue studies have revealed that multiple cell types, including germ cells, Sertoli cells, Leydig cells and testicular resident macrophages (CD163+), are permissive to infection [5,6,7]. However, ZIKV antigens have never been found in ejaculated sperm cells.

There are several case studies and case-series of travelers returning from endemic areas to European countries or USA reporting sexually-acquired ZIKV infection, mainly by transmission from infected males to uninfected females (94%), following vaginal intercourse [8,9,10,11,12]. Some of these studies have shown that a substantial proportion (47–56%) of males who had a diagnosis of imported ZIKV infection had detectable ZIKV RNA in semen during convalescence [13,14]. There is limited evidence of ZIKV in semen from individuals living in countries affected by Zika, but one study reported shedding in 33% of the ZIKV-infected male within a time frame of 14 to 304 days postsymptoms onset (DPSO) [12]. ZIKV shedding in semen was limited to 30 DPSO in the majority of those individuals (61%). Studies on the kinetics of ZIKV RNA in semen showed viral loads in the range of 1 × 10^2^ to 1 × 10^9^ genome copy equivalents/mL [15,16]. High copy numbers of ZIKV RNA have been also reported in semen from asymptomatic blood donors who previously tested positive for ZIKV RNA in plasma (8 × 10^3^ to 1 × 10^6^ copies/mL) [17]. The reported isolation of infectious particles in the seminal plasma and the motile fraction [9,12] is suggestive of potential sexual transmission. Studies showing high viral load and long-term ZIKV shedding in semen also described low viral load and short-term detection in blood [18], suggesting a possible reservoir of replication in the male reproductive tract, rather than migration from blood [17,18,19]. Antisense ZIKV RNA, a replication intermediate, has been detected in semen collected within 21 DPSO (Ct < 26) from men diagnosed with ZIKV infection [14], suggesting active infection. Altogether, these studies suggest that unlike most other flaviviruses [20], ZIKV can be sexually transmitted and may persist for up to nine months in the male reproductive tract [12,13,21,22,23,24], thereby presenting a new challenge for transmission and reproductive medicine in endemic regions [12,14,15,22,23,24].

Very few studies have described the presence of ZIKV in semen from asymptomatic subjects [17], but a high prevalence (56–78%) of asymptomatic infections in the general population of affected countries has been demonstrated in serological studies [25,26]. By examining semen from Nicaraguan men during and shortly after the introduction of ZIKV to the country, we address the hypothesis that ZIKV may exhibit prolonged shedding from the male reproductive tract in symptomatic and asymptomatic subjects, potentially contributing to endemic transmission and underlying fertility issues in some men. The study was conducted during the 2016–2017 ZIKV epidemic, during which approximately 60% of the population of León, Nicaragua was infected with ZIKV [25].

## 2. Materials and Methods

### 2.1. Study Design and Population

During an observational study of acute ZIKV infections in León, Nicaragua, carried out between November 2016 to February 2017, a total of 16 adult male patients were confirmed to have acute ZIKV infection by RT-qPCR analysis of blood, saliva and urine. Of these patients, three agreed to participate in a longitudinal study of ZIKV shedding in semen. The cases were compared to a convenience sample of asymptomatic subjects from the same area (*n* = 19), which reported not experiencing Zika-like symptoms in the last month and never having been diagnosed with Zika. Both cases and asymptomatic subjects lived in the urban area of León, Nicaragua, and were enrolled within the same time frame. Cases collected a single semen sample at 7, 14, 21, 28, 60, 90 and 180 DPSO, and asymptomatic subjects donated one semen sample at enrollment only. Semen samples were collected by masturbation at the andrology laboratory at the Medical Campus in León. An aliquot of each semen sample was stored at −80 °C for cell culture analysis. All subjects also provided a blood sample for ZIKV antibodies and RT-qPCR analysis. All subjects provided written informed consent and provided clinical and epidemiological information related to febrile illnesses. This study was reviewed and approved by the local ethical committee for biomedical research (UNAN-León, Acta No. 85, 2016, FWA00004523).

### 2.2. Examination of Conventional Semen Parameters

Semen samples were examined within an hour after collection by following the 2010 World Health Organization laboratory manual for the examination of human semen, 5th edition [27]. The following parameters were examined: color, liquefaction time, volume, viscosity, pH, concentration, motility, vitality, round cells count and Kruger strict morphology.

### 2.3. Semen Preparation for Immunofluorescence (IF)

To separate seminal plasma from sperm cells, 100 µL of semen was added to a vial containing 900 µL of phosphate buffered saline, pH 7.2 (PBS), and gently mixed to remove sperm cells clumps. After 10 min of centrifugation at 1500 rpm, the supernatant was removed and stored −80 °C until RT-qPCR analysis. To remove any remaining seminal plasma, the sperm cell pellet was washed with 900 µL of PBS, centrifuged, and the supernatant was discarded. Finally, the pellet was resuspended with another 900 µL of PBS for storage −80 °C until IF and RT-qPCR analysis.

### 2.4. Immunofluorescence (IF) for Detection of Flavivirus Antigens

To investigate whether the 4G2 monoclonal antibody recognized ZIKV, Vero-E6 cells infected with the ZIKV strain H/PF/2013 and cultivated for five days were examined by immunofluorescence. To identify Flavivirus antigens in sperm cells, 15 µL of the sperm suspension was added in duplicate to Superfrost^®^ Plus slides (Thermo Scientific, Waltham, MA, USA), dried at room temperature (RT) and fixed with para-formaldehyde solution (4% in PBS) at 4 °C for 5 min, followed by washing with PBS for 5 min at RT. For the detection of Flavivirus antigens, 30 µL of 1:200 antiflavivirus monoclonal 4G2 [28] was added to each well and incubated at 37 °C for 30 min in a humid chamber. After washing three times with PBS, for 5 min each time, 30 µL of 1:200 goat antimouse IgG conjugate (Alexa Fluor 488, Thermo Fisher Scientific, A-11001) was added and the mixture was incubated at 37 °C for 30 min in humid chamber in the dark. The slides were washed, and 30 µL (5 µg/mL) of DAPI (4′,6-diamidino-2-phenylindole, Thermo Fisher Scientific, D1306) was added as a nuclear counterstain for fluorescence microscopy. After 2 min of incubation, slides were washed and mounted with fluorescent mounting medium (Dako, S3023, Santa Clara, CA, USA). Slide scanning was performed with 40× objective in the Nikon Labophot-2 Microscope (green filter, Badhoevedorp, The Netherlands). A sample was considered positive if at least one cell with sperm-like morphology contained bright green spots after scanning 10 high power fields with 40× objective and confirmed with 100× objective. All specimens were examined in duplicate. The total number of sperm cells in the scanned ten fields was manually counted by using the DAPI filter (UV-2E/C DAPI) and 40× objective. For quality control, slides with positive immunofluorescence were transferred to Prof. Lennart Svensson laboratory in Sweden for scanning with an inverted confocal microscope (ZEISS LSM700, Oberkochen, Germany) and pictures were taken with 63× objective. The same IF analysis were performed in the cell culture inoculated with semen samples.

### 2.5. RNA Purification

Viral RNA was extracted from 140 µL of sperm cell suspensions and supernatant using the QIAamp viral RNA minikit (Qiagen, Hilden, Germany) according to the manufacturer’s instructions. A total of 60 µL of RNA was collected and stored at −20 °C for ZIKV detection by RT-qPCR.

### 2.6. Reverse Transcriptase and Quantitative Polymerase Chain Reaction (RT-qPCR)

ZIKV RNA was detected by using 2 primers/probe amplification sets specific for ZIKV and AgPath-ID™ One-Step RT-qPCR reagent (Applied Biosystem, Waltham, MA, USA). One primer set detects all known genotypes of ZIKV and the second is specific for the Asian genotype that circulated in the Western Hemisphere [29]. In brief, 8 µL of RNA was added to a reaction mixture consisting of 12.5 µL of buffer 2× RT-qPCR, 1 µL (10 pmol) of each Zika1087 and Zika1163c primers and 1 µL (10 pmol) of Zika1108-FAM probe, 1.5 µL of RNAse free water, to final volume of 25 µL. For the second reaction the following primers and probes were used: Zika4481, Zika4552c and Zika4507c-FAM. The RT-qPCR reactions were performed in a 96-well reaction plate using the Applied Biosystems^®^ 7500 Real-Time PCR Systems. RT-qPCR was performed under the following conditions: 50 °C for 30 min, 95 °C for 15 sec, followed by 40 cycles of 95 °C for 1 min, 60 °C for 1 min. Any sample with a cycle threshold (Ct) of ≤38 with a cut off of 0.05 was considered positive, in accordance with the method described by Lanciotti and coworkers [29]. In this study, a specimen was considered positive if either reaction showed a Ct ≤38. In every batch of reactions, a nontemplate control was included and the supernatant of the ZIKV FP strain was used as positive control. Cases were further confirmed by using the Trioplex (DenV, ZIVK and ChikV) RT-qPCR assay from CDC.

To avoid analysist bias, samples codes were blinded for analyst and the IF and RT-qPCR assays were performed by independent researchers, respectively.

### 2.7. PCR for E-Gene Amplification and Sequencing

In brief, the PCR mix contained 5 µL of cDNA, 1 µL (10 pmol) of each forward (ZIKAENVF) and reverse (ZIKAENVR) primers (IDT. Inc, Coralville, IO, USA) [30], 18 µL of RNAse free water and 1 PuReTaq Ready-To-Go PCR bead (GE Healthcare Bio-Sciences AB, Uppsala, Sweden). The RT-PCR reaction was performed in the Applied Biosystems ABI 2720 Thermal Cycler (Foster City, California, USA) under the following conditions: 95 °C for 2 min, followed by 35 cycles of 95 °C for 20 s, 55 °C for 20 s, 68 °C for 30 s, with a final extension of 68 °C for 7 min. Gel electrophoresis was performed to identify an amplicon of 364 bp, indicating the presence of ZIKV RNA. The PCR products were sequenced in the forward and reverse direction by Macrogen Europe (Amsterdam, the Netherlands), and basic local alignment search tool (BLAST) analyses were performed to determine the nucleotide homology with available sequences in the GenBank. The phylogenetic tree was constructed based on neighbor-joining method by using the ZIKV typing tool [31].

### 2.8. Flavivirus IgG and IgM Detection

To investigate if ZIKV PCR-positives were recently infected, anti-ZIKV IgG and IgM were measured in acute (at presentation) and convalescent (28 DPSO) serum of cases, and in the serum collected from controls at presentation. IgG and IgM antibody capture ELISA were used as described Collins and coworkers [32] and the CDC MAC ELISA [29], respectively. Acute flavivirus infection was defined by the presence ZIKV. For asymptomatic subjects, the presence of ZIKV IgM in the serum collected at presentation was indicative of recent flavivirus infection. Due to the cross reactivity between ZIKV and DenV antibodies, the data presented refer to flavivirus serology.

### 2.9. Virus Isolation

The semen samples collected from patient A at 14 and 21 DPSO were inoculated in cell culture to investigate infectivity of the complete semen or their fractions (seminal plasma and cells). In brief, 200 µL of neat semen, 500 µL of seminal plasma or 500 µL of cell suspension were diluted in 2 mL of DMEN supplemented with 2% FBS, then inoculated in a flask (F25) of confluent Vero cells monolayer and incubated for 1 h at 37 °C, with 5% CO_2_ and 95% humidity, followed by addition of 5 mL of fresh media. Cytopathic effects were examined daily for five days of incubation, after which the supernatant was passed to a new monolayer. The same procedure was conducted after inoculation with the ZIKV strain H/PF/2013.

### 2.10. Statistical Analysis

Laboratory data were analyzed by SPSS (version 21, IBM, New York, NY, USA) to perform basic statistical calculations. Student’s t-tests for independent samples were performed to compare mean values of the semen parameters from specimens collected from ZIKV-positive and ZIKV-negative subjects.

## 3. Results

### 3.1. Flavivirus Antigens Were Detected in Sperm Cells from Symptomatic Subjects

**Figure 1:**
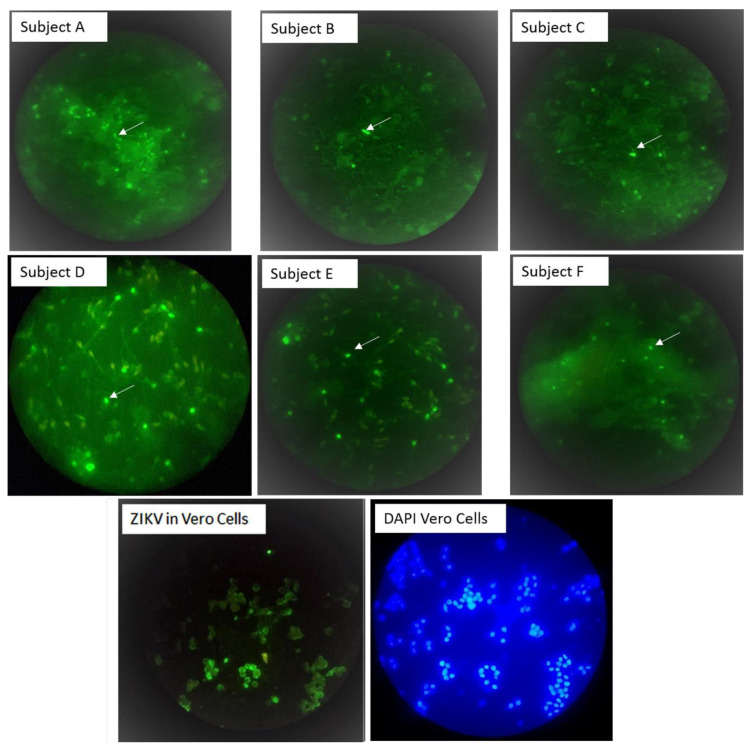
Zika virus binds to sperm cells. Sperm IF staining with the flavivirus 4G2 monoclonal antibody and Alexa fluor-488 conjugate (green). Subjects A, B and C were patients with Zika symptoms, and subjects D, E and F were asymptomatic controls. A sample was considered positive if at least one cell with sperm-like morphology contained bright green spots (see white arrows) after scanning ten high power fields with 40X objective in duplicates samples. The image of ZIKV in vero was performed to evidence that 4G2 is capable of recognizing ZIKV. All images were taken with 40× objective.

Flavivirus antigens were detected in sperm from all three (100%) of the cases. These subjects (A, B and C) were also shown to be ZIKV-positive by RT-qPCR in either blood, saliva or urine samples collected during the acute phase of infection (Table 1). These subjects were re-examined for ZIKV, DenV and ChikV by Trioplex RT-qPCR analysis and confirmed as ZIKV-positive, DenV-negative and ChikV-negative. The three patients provided 12 subsequent semen samples, of which six (50%) were RT-qPCR-positive at either 7, 14, 21 or 28 DPSO; two out of five from subject A, three out of five from subject B and one out of two from subject C (Table 1). Flavivirus antigens were detected in 5 (42%) of the 12 semen examined, i.e., on average, 2.4% of the spermatocytes (range 1.9% to 25%) were ZIKV IF positive per total cells counted in each of these samples (Table 1, Figure 1. Panel A, B and C). ZIKV IgM and IgG analyses of acute and convalescent serum samples confirmed recent flavivirus infection in the symptomatic subjects A and B. ZIKV ARN was detected at 180 DPSO from subject A, but this result could not be replicated in further RT-qPCR analysis.

### 3.2. Flavivirus Antigens Were Found in Asymptomatic Subjects

Flavivirus antigens and RNA were also detected in sperm from 3 (16%) of 19 asymptomatic men enrolled during the period of high ZIKV transmission in León, Nicaragua (Table 1). ZIKV IgM analysis of serum samples collected at enrollment confirmed recent flavivirus infection in one out of the three asymptomatic individuals (subject F). The percentage of ZIKV IF-positive cells per total cells counted (with DAPI staining) in ten fields scanned ranged from 0.8% to 1.4% in these three asymptomatic individuals (Table 1, Figure 1, panel D, E and F). While ZIKV RNA was detected in sperm cell suspension, all semen supernatants were ZIKV RNA-negative according to the RT-qPCR results.

### 3.3. Correlation between Antigen and RNA Detection

Poor correlation was observed after IF and RT-qPCR analysis of samples collected at the same time point. For instance, none of the seven serial samples collected from subject A was positive with both methods; this is suggestive of the presence of PCR inhibitors in the semen of that individual, or mutations in the region of the genome targeted by our PCR assay (Table 1).

### 3.4. Immunofluorescent Patterns

**Figure 2:**
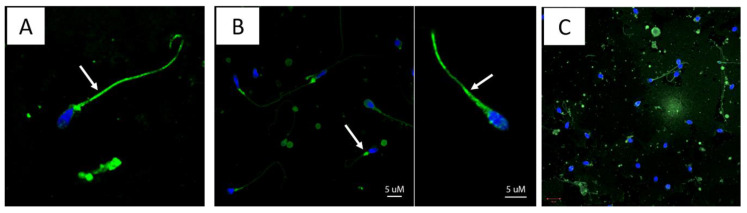
Immunofluorescent patterns. Slides with IF-positive (green) re-examined with confocal microscopy. Figures in this panels shows that Flavivirus antigens (green, Alexa 488) binds to sperm cells, with high affinity to the tail (**A**,**B**) and the neck (**B**) of the sperm, as pointed out by white arrows. Green spots were also observed in the head of some sperms as shown in **C** (center). Pictures were captured using confocal microscopy (LSM700 Zeiss, Oberkochen, Germany). All images were taken with 63× objective.

To explore IF-patterns in more details, the IF-positive samples from subject A and E were also scanned with confocal microscopy, showing sperm cells with marked IF-pattern in the cytoplasmic droplets and tail; IF staining was also observed in the sperm head of some cells (Figure 2, panel A, B and C).

### 3.5. Virus Isolation

Vero E6 cell infection with semen from the subject A (specimens collected at 14 and 21 DPSO), which shows a high percentage of cells with IF, did not show cytopathic effect (passage 3). The cell pellet was IF-negative but RT-qPCR of the supernatant was positive (Ct = 36).

### 3.6. Partial Sequence Analysis of the E Gene

The consensus of the forward and reverse sequences (293 bp) segment from the *E* gene (subject B) shows ≥98.6% of nucleotide identity with E (1595 to 1887) sequence from the “H/PF/2013” strain and several other sequences of ZIKV from South and Central America. The Nicaraguan sequence was assigned to the Asian clade using the Zika Virus Typing Tool (Figure S1).

### 3.7. Correlation between Fresh Semen Analyses of Subjects ZIKV-Positive and ZIKV-Negative

To explore if semen quality is affected by ZIKV infection either symptomatic or asymptomatic, spermiogram parameters of specimens collected from the ZIKV-positive subjects (*n* = 13; ten from the three cases and three asymptomatic subjects) and ZIKV-negative subjects (*n* = 16, from asymptomatic) were compared (Table 2). The median age of the ZIKV-positive and ZIKV-negative was 21.5 (range 17–42) and 21 years (range 18–24), respectively, and the median days of abstinence was 5 days in both groups. The sperm concentrations (45 × 10^6^/mL vs. 63.5 × 10^6^/mL, *p* = 0.041) and the total motility percentage (54% vs. 75%, *p* = 0.009) was significantly lower in specimens from ZIKV-positive than in those of ZIKV-negative, respectively. Regarding Kruger strict morphology, we observed 2% of normal morphology in ZIKV-positive as compared with 3% in ZIKV-negative, with a great percentage of sperm with head defects in specimens from ZIKV-positive (42% vs. 16%, *p* = 0.054) (Table 2).

## 4. Discussion

This study builds upon other studies confirming that ZIKV RNA is frequently present in the male reproductive tract within 30 DPSO [12,14,16] and provides new evidence that sperm cells are able to harbor Flavivirus antigens. These data also complement a preliminary report indicating that asymptomatic blood donors may shed RNA in semen after symptom resolution [17]. Moreover, the current study demonstrates that the presence of Flavivirus antigens in spermatocytes from ZIKV-positive subject correlates with variations in several parameters of the spermiogram. These findings support the hypothesis that ZIKV can infect sperm cells, having a direct cytopathic effect on sperm, with temporal implications for male fertility.

The current study performed in Nicaragua demonstrated the presence of Flavivirus antigens and RNA in sperm cells of three symptomatic subjects within 30 DPSO, with the E partial nt sequence from one subject being highly homologous with sequences from the ZIKV Asian clade, including the French Polynesia strain and other reported sequences of ZIKV circulating in the Caribbean and Central America at the same time frame, suggesting a role of viral genetic determinants on sexual transmission.

Similar observations were reported in the French Caribbean island of Guadeloupe, with 4 (27%) our of 15 symptomatic patients shedding ZIKV RNA in semen within 20 DPSO and evidence of infectious virus, as measured by RNA increase in cell culture in specimens from one subject [9]. Extended follow-up of the cohort from Guadeloupe showed evidence of ZIKV RNA in semen until 414 DPSO, albeit with very low RNA copies (1 × 10^2.4^ RNA copies) [9,33]. Another study from Puerto Rico, which followed a similar sampling protocol but included more (*n* = 55) symptomatic subjects, showed that 51% of symptomatic men shed ZIKV RNA in semen; the median time until RNA clearance was 34 DPSO (95% CI, 28 to 41 DPSO), with 4% of participants shedding ZIKV RNA up to 125 DPSO [10]. A common characteristic of the current and the other two cohorts was the observation of intermittent RNA detection in some individuals, suggesting PCR inhibition. The observation of intermittent antigen detection in the current study (see subject C) could be further explored in the context of sexual abstinence and more consistent sampling. For couples planning pregnancy, serial sampling will be necessary to confirm ZIKV clearance in semen.

An interesting observation in the current study was the fluorescence pattern observed in the cytoplasmic droplet and sperm tail from specimens collected from subjects A, C and E (Figure 2, panels A, B and C), suggesting that the virus remains attached to the sperm. Whether these viruses retain the infectious capacity and spermatozoa can carry these viruses to a new infectious niche remain to be fully investigated. There is some evidence, however, that ZIKV in semen can be infectious in cell culture; the Puerto Rican study showed that 6 out of 20 semen specimens with low Ct (19 to 27) infected vero cells, and the Guadeloupe study described virus isolation from the seminal plasma and spermatozoa fractions of one patient with high RNA copies (10^10^ at 7 DPSO) [9,10]. Müller and coworkers showed that semen inhibits ZIKV infection of cells lines, including Vero E6, by preventing attachment to target cells; an extracellular vesicle preparation from semen was responsible for this anti-ZIKV activity [34]. Altogether, these observations suggest that ZIKV transmission by semen may be less efficient than mosquito transmission. Whether lipid droplets of the spermatozoa function as vehicles for ZIKV transmission, or semen plasma composition contributes to overcoming the mucosal immunity in the vagina, remain to be investigated.

Among the spermiogram parameters examined, lower sperm concentration, total motility percentage and a greater percentage of head defects were observed in ZIKV-positives compared to ZIKV-negative. These findings correlate with the observations of Joguet and coworkers, describing multiple sperm anomalies in ZIVK-positive subjects [9]. Increased leukocytospermia, hematospermia and oligospermia were also reported by Huits and coworkers in a cohort of ZIKV-infected travelers returning to Belgium [35]. Altogether, our and other reported data suggest that ZIVK infection reduces sperm quality and likely affects male fertility. However, such trends should be examined further in larger datasets, as the analyses from the current study were based on specimens collected from only six ZIKV-positive subjects. An interesting observation was the presence of green fluorescent cell debris in the semen of ZIKV RNA-positive subjects (Figure 2, panel A); whether such debris carries the ZIKV E viral protein or cross reactive antigens remains to be investigated.

The consequences of ZIKV infection in the male reproductive tract was explored in mice (treated with anti-Ifnar Mab) by Govero and coworkers. Those authors indicated that ZIKV preferentially infected spermatogonia, primary spermatocytes, and Sertoli cells in the testis, resulting in cell death and the destruction of the seminiferous tubules [3]. Another study showed that primary human testicular tissue, in particular, human germ cells, were susceptible and permissive for ZIKV infection [5].

In conclusion, this study found that Flavivirus antigens and Zika RNA can be present within a time frame of 28 DPSO in the semen of symptomatic and asymptomatic subjects during ZIKV epidemics. Such findings have implications for male fertility and public health, considering that ZIKV infection may decrease semen quality, and long-term shedders might contribute to silent viral spread through sexual contact. Moreover, the transmission of ZIKV to a pregnant female partner might have serious consequences for the fetus.

The total number of symptomatic individuals, the use of specific flaviviruses instead of ZIKV-specific Mabs and the use of conventional IF microscopy for antigen screening are limitations for making definitive statements, but these limitations in aggregate do not invalidate the conclusions of this study.

## Figures and Tables

**Table 1:** Description of the clinical status and detection of Flavivirus antigens and RNA in spermatozoa from subjects exposed to the virus during the emergency in Nicaragua in 2016–2017.

Subjects ^a^	Clinical Status	Age (Years)	Date of Enrollment	Flavivirus Serology ^b^	DPSO ^c^	ZIKV Antigen in Sperm Cells by IF	% of IF Positive Sperm Cells	ZIKV RNA in Sperm Cells (Ct1/Ct2) ^e^
A	Symptomatic	21	Nov/2016	IgM-positiveIgG-negative	7	Yes	3 (11/337)	No
					14	Yes	2.4 (18/737)	No
					21	Yes	25 (321/1266)	No
					28	No	0 (0/663)	Yes (21/40)
					180	No	0/1603	Yes 40/31
B	Symptomatic	22	Nov/2016	IgM-positiveIgG-positive	7	Yes	4.3 (4/916)	Yes (22/21)
					14	No	0 (0/226)	Yes (32/36)
					21	No	0 (0/723)	Yes (32/40)
					28	No	0 (0/370)	No (40/40)
					60	No	0 (0/507)	No (40/40)
C	Symptomatic	43	Aug/2017	IgM-inconclusive	21	No	0 (0/486)	No (40/40)
				IgG-positive	28	Yes	1.9 (18/935)	Yes (22/24)
D	Asymptomatic	21	Jan/2017	IgM-negative	NA ^d^	Yes	0.8 (4/487)	Yes (33/35)
				IgG-positive				
E	Asymptomatic	22	Jan/2017	IgM-negative	NA	Yes	1.4 (10/724)	Yes (40/37)
				IgG-positive				
F	Asymptomatic	19	Jan/2017	IgM-positive	NA	Yes	3.8 (18/472)	Yes (40/36)
				IgG-positive				

^a^ After initial screening by RT-qPCR of blood, saliva and urine, subject A was positive in saliva only, B was positive in blood only and C was positive in urine only. Further triplicated analysis confirmed these results. ^b^ Only ZIKV IgG and IgM were examined; the data is presented as flaviviruses serology due to cross reactivity with DenV. Positive stands for ≥4-fold OD increase between acute and convalescent serum. ^c^ DPSO = Days Post Symptoms Onset; ^d^ NA = Not Apply. ^e^ Cycle threshold 1(Ct1) stands for the primers set that detects all known ZIKV genotypes and Ct2 for the set specific for the Asian genotype [29].

**Table 2:** Comparison of semen parameters in specimens collected from ZIKV-positive and ZIKV-negative subjects according to the 2010 World Health Organization laboratory manual for the examination of human semen.

Semen Parameters	Zika Immunofluorescence		*t*-Test, *p*
	ZIKV-Negative (*n* = 16)	ZIKV-Positive (*n* = 14)	
Median age in years (min and max)	21.5 (17 and 42)	21 (18 and 24)	0.999, (0.330)
Median of semen volume (ml)	2.3 (IQR 1.1–3)	2.5 (IQR 2–3.5)	−1.031, (0.359)
Median of the sperm concentration (million/mL) ^a^	63.5 (IQR 36–104)	45 (IQR 27–54)	2.4 (0.041)
Mean motility (%, range)			
Total motility (%, range) ^b^	75 (50–100)	54 (27–90)	2.883 (0.009)
Progressive motility (%, range)	60 (30–80)	46 (20–81)	2.020, (0.063)
Nonprogressive motility (%, range)	15 (0–30)	8 (1–20)	1.99, (0.050)
Nonmotile ^b^ (%, range)	25 (0–65)	46 (10–73)	−2.870, (0.009)
Mean viability (%, range)	86 (60–98)	72 (10–98)	1.492, (0.159)
Normal morphology (%, range)	3 (0–15)	2 (0–9)	0.456, (0.656)
Abnormal distributions (%, range)			
Head defects (%, range)	16 (0–100)	42 (0–86)	−2.043, (0.051)
Neck mid-piece defects	5 (0–16)	6 (0–51)	0.112, (0.909)
Tail defects	8 (0–34)	7 (0–25)	−0.431, (0.674)
Excess residual cytoplasm	11 (0–66)	5 (0–35)	−0.936, (0.369)
Median of the round cells concentration (million/mL)	4.8 (IQR 2.7–10.6)	5.1 (IQR 0.6–7.7)	1.1 (0.286)

^a^ This parameter was not evaluated in three specimens collected from a ZIKV-positive subject because of increased semen viscosity and incomplete liquefaction; a one-way ANOVA analysis confirmed significant differences in this parameter (F = 5.9, *p* = 0.02); ^b^ One way ANOVA analysis confirmed significant differences in these two parameters.

## Data Availability

The data presented in this study are available on request from the corresponding author. The data are not publicly available due to limitations in material transfer agreement.

## Supplementary Materials

The following are available online at https://www.mdpi.com/1999-4915/13/2/152/s1, Figure S1: Phylogenetic analysis of a partial sequence from the E ZIKV gene.
