# Supplementary material for: Zika RNA and Flavivirus-Like Antigens in the Sperm Cells of Symptomatic and Asymptomatic Subjects
Source: Viruses. 2021 Jan 21;13(2):152. doi: 10.3390/v13020152 (PMC7909808; doi:10.3390/v13020152)
Supplement: Supplementary file 1 [file viruses-13-00152-s001.pdf]

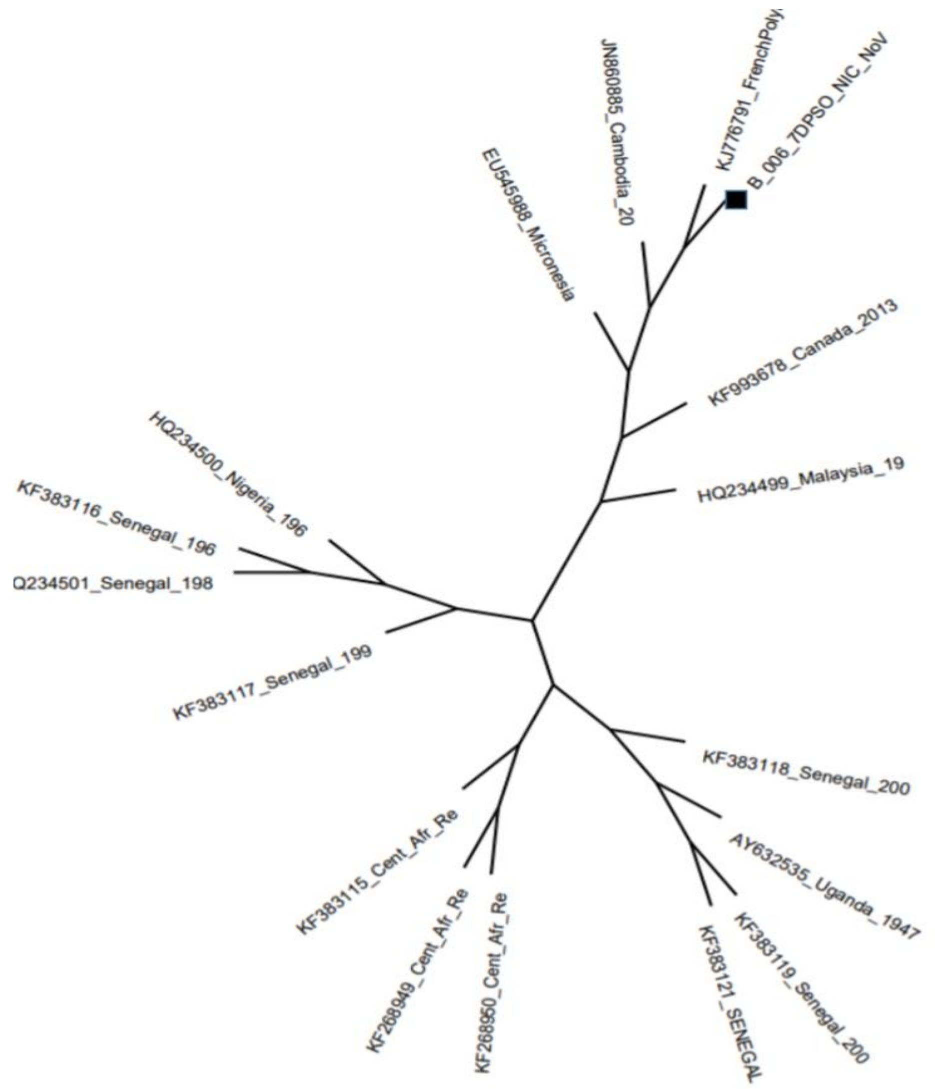

Figure S1. Phylogenetic analysis of a partial sequence from the E ZIKV gene. The consensus of the forward and reverse sequences (293 bp) segment from the E gene (nt 1595 to 1887, ref KJ776791). The phylogenetic tree was constructed based on neighbor-joining method by using the ZIKV typing tool Ref [31]. The black square represent the Nicaraguan sequence (Subject B, 7DPSO, NIC, November 2016).
